# Evaluation of pathways and new host proteins involved in CD4 down-modulation during HIV-1 infection

**DOI:** 10.1186/1742-4690-8-S2-P43

**Published:** 2011-10-03

**Authors:** Alessia Landi, Mostafa Bentahir, Jolien Vermeire, Hanne Vanderstraeten, Veronica lanucci, Bruno Verhasselt

**Affiliations:** 1 Department of Clinical Chemistry, Microbiology, and Immunology, Ghent University, Belgium; 2 Present address: Centre de Technologies Moléculaires Appliquées, Ecole de Santé Publigue, Brussels, Belgium

## 

Down-modulation of the CD4 receptor expressed on T cells is one of the hallmarks of Human Immunodeficiency Virus (HIV) infection and it is believed to confer to the virus a selective replicative advantage in vivo. HIV has evolved redundant mechanisms to remove the receptor from the cell surface and accelerate its degradation. This process is mainly mediated by three viral proteins: Vpu, Env and Nef. Up to date, the mechanisms that lead to CD4 depletion from the surface of CD4+ T lymphocytes, the natural targets of HIV, are still poorly understood and only partially characterized. We are interested in the discovery of pathways and human proteins involved in the process, in order to eventually find potential new drug targets. To pursue our aim, we first performed a functional screening on HeLa CD4+ cells expressing Nef, using a shRNA lentiviral interference delivery system targeting the whole human genome compatible with an Affymetrix GeneChip Microarray. The read out was the rescue of the CD4 high phenotype, despite Nef expression. The results were analyzed with Affymetrix Expression Console 1.1 software and the web-based gene ontology resource DAVID. After four different screens we obtained a final list of 75 genes enriched in the cells sorted for high surface CD4. These genes appear to code for proteins involved in endocytic trafficking, trans-Golgi trafficking and lysosomal degradation pathways. We decided to validate our hits in primary blood CD4+ T cells, the most relevant for this kind of studies, and we set up a protocol for the transduction of these cells with lentiviral and retroviral vectors, necessary respectively for the expression of the shRN As and the viral proteins Vpu, Nef and Env. The actual down-regulation of the gene expression will be validated via quantitative Real Time PCR and Western Blot and the experiments will be eventually repeated in the context of HIV-1 infection.

